# Multi-task topology optimization of photonic devices in low-dimensional Fourier domain via deep learning

**DOI:** 10.1515/nanoph-2022-0361

**Published:** 2022-10-04

**Authors:** Simei Mao, Lirong Cheng, Houyu Chen, Xuanyi Liu, Zihan Geng, Qian Li, Hongyan Fu

**Affiliations:** Tsinghua-Berkeley Shenzhen Institute and Tsinghua Shenzhen International Graduate School, Tsinghua University, Shenzhen 518055, China; School of Electronic and Computer Engineering, Peking University, Shenzhen 518055, China

**Keywords:** deep learning, inverse design, silicon photonics, topology optimization

## Abstract

Silicon photonics enables compact integrated photonic devices with versatile functionalities and mass manufacturing capability. However, the optimization of high-performance free-form optical devices is still challenging due to the complex light-matter interaction involved that requires time-consuming electromagnetic simulations. This problem becomes even more prominent when multiple devices are required, typically requiring separate iterative optimizations. To facilitate multi-task inverse design, we propose a topology optimization method based on deep neural network (DNN) in low-dimensional Fourier domain. The DNN takes target optical responses as inputs and predicts low-frequency Fourier components, which are then utilized to reconstruct device geometries. Removing high-frequency components for reduced design degree-of-freedom (DOF) helps control minimal features and speed up training. For demonstration, the proposed method is utilized for wavelength filter design. The trained DNN can design multiple filters instantly and concurrently with high accuracy. Totally different targets can also be further optimized through transfer learning on existing network with greatly reduced optimization rounds. Our approach can be also adapted to other free-form photonic devices, including a waveguide-coupled single-photon source that we demonstrate to prove generalizability. Such DNN-assisted topology optimization significantly reduces the time and resources required for multi-task optimization, enabling large-scale photonic device design in various applications.

## 1 Introduction

Integrated photonics has attracted enormous attention for its merits of low power consumption and high bandwidth. What is more, silicon photonics is compatible with complementary metal oxide semiconductor (CMOS) process, which enables massive fabrication of integrated photonic devices at low cost. Silicon photonic devices and systems have been widely used for optical communications [1], optical computations [2] and quantum optics [3]. Unlike electric circuits which realize different functions through the combination of basic electronic components, a single photonic device can achieve versatile functions by designing device structures. However, due to the complex light–matter interaction, the design of specific-functional devices is not explicit. Especially for those devices with irregular structures, their performance evaluations have to rely on time-consuming numerical electromagnetic (EM) simulations [4], such as the three-dimensional finite-difference-time-domain (3D FDTD) method.

To facilitate the design silicon photonic devices, various inverse design methods based on EM simulations have been proposed, like heuristic algorithms and gradient-decent algorithms [5, 6]. Heuristic algorithms, such as genetic algorithms [7–9] and particle swarm optimization [10–12], generate a group of initial design parameters and update them towards the direction with larger figure-of-merit (FOM). Heuristic algorithms are applicable when the design degree-of-freedom (DOF) is fairly low (less than hundreds), as the required simulations at each epoch are supposed to be larger than the DOF at each iteration. The gradient-decent algorithms like the adjoint method are suitable for designing devices with large DOF (over thousands), where all design parameters could be updated with two simulations [13–15]. However, the gradient-decent algorithms are prone to fall into local optimal [6, 16]. The minimal feature size control is also a big issue, especially for topology optimization (TO) where all pixels in structure are taken as DOF. Some researchers try to solve it by adding a penalty term to loss function [17], applying a filter to remove small features at each iteration [18] or enlarging the size of each pixel to above critical values [19]. Recently, the re-parameterization methods have also been proposed to control the minimal feature size [20].

The above iterative algorithms are more suitable for the single-task optimization. When the target is varied, the whole tedious optimization procedure has to be performed again. Thankfully, deep neural networks (DNNs) bridging the relationship between input and output have been successfully applied for the design of silicon photonic devices [21–27]. However, there are two challenges for DNN-based inverse design methods. Firstly, the DNN requires a great size of prepared training data samples with high performance, usually a thousand times larger than DOF, which is extremely time-consuming to generate. Melati et al. proposed to use machine learning to map the high-dimensional original design space to sub-space with lower dimensionality [28]. However, this method also requires lots of training data samples for this mapping process. Liu et al. proposed a transform-domain-based encoding method to compress real space image into sparse representation in Fourier domain without the dependance of data [29]. However, it uses rigorous Fourier domain processing so that conjugate symmetry is preserved in the post-processing Fourier spectra. Even though the images are restored rigorously, some potential images, are sacrificed due to the selective choice of low-frequency components. Secondly, the trained DNN has limited generalizability. If a target is too far away from the training data set, the network may lose its ability for prediction. Jiang et al. proposed a simulator-based DNN training method for global optimization of 1D metagratings [16, 30], where no prepared training data sample is required. Nevertheless, it is limited to optimization of 1-D periodic structures, whose design DOF is as low as 25 and the dimension of optical target is only 2. What’s more, the EM simulation time for each device is less than one second, already allowing quick optimization by other heuristic algorithms as demonstrated previously [5–15]. In contrast, efficient optimization of 2D free-form device is in greater need, due to the long simulation time and high DOF of 2D free-form structures.

In this work, we propose a DNN-assisted multi-task topology optimization method in Fourier domain with minimal feature size control for 2D free-form devices. The DNN maps optical responses to low-frequency components in Fourier domain, while the topology optimization serves as a supervisor to guide the training of DNN. The device structures can be reconstructed by inverse fast Fourier transform (IFFT) from Fourier domain. Here, we only take the low-frequency components as DOF instead of all pixels in real space, because images in real space contain high-frequency components that contribute to unwanted noise and details. In this way, minimal features in the device structure can be reduced. Another advantage of this Fourier transform domain processing is that it reduces redundant DOF and makes network training easier to converge. For proof-of-concept demonstration, we apply this novel method for the design of integrated wavelength filters on silicon-on-isolator (SOI) platform. Multiple randomly generated filtering targets can be achieved with high accuracy using our multi-task optimization. The trained DNN can be used to predict the wavelength filters with high accuracy instantly. What is more, the trained DNN can then be used to further design other filters with totally different spectra through transfer learning. The proposed method can also be used for multi-stage optimization, with simple DNNs requiring reduced computation resources at each stage. To further demonstrate the generality of our inverse design procedure, we adapt it to the design of on-chip single photon source with high waveguide coupling efficiency achieved.

## 2 Theoretical analysis

### 2.1 Topology optimization with adjoint method

For a target optical response ***y*** like the low-pass, high-pass, band-pass or band-stop spectra, the image pattern **X** needs to be specified through optimization. The proposed device structure has 201 × 101 pixels in total, i.e., the design DOF of the device is 20,301, which is hard to optimize by heuristic algorithms. Fortunately, topology optimization with adjoint method is capable of processing such design with numerous DOF. The optical response ***y*** is the analytical function of electric fields **E** distributions at the output port, given as

(1) $$y=f_{1}\left(E\right),$$

where the electric field distributions **E** in the output area is determined by the light–material interaction in the design area. For a normalized injection light source, the output electric filed **E** is varied with the permittivity ***ɛ*** at the design area, given as

(2) $$E=f_{2}\left(\varepsilon \right).$$

The function *f*_2_ is an implicit function, which transfers the light-material interaction from the design structure to the output area. The derivative of **E** with respect to permittivity ***ɛ*** can be calculated by the adjoint method [31] as

(3) $$\frac{dE}{d\varepsilon }=2\varepsilon_{0}dVRe\left[E_{\text{fwd}}\cdot E_{\text{adj}}\right],$$

where *ɛ*_0_ is the vacuum permittivity and d*V* is the volume of each pixel. **E**_fwd_ and **E**_adj_ are electric fields at the design area calculated by forward and inverse simulations. Supposing the permittivity of silica and silicon are *ɛ*_1_ and *ɛ*_2_, the *ɛ*_*ij*_ is represented by the grayscale value of pixel *x*_ij_ in the design area as

(4) $$\varepsilon_{ij}=\varepsilon_{1}+\frac{x_{ij}+1}{2}\left(\varepsilon_{2}-\varepsilon_{1}\right).$$

For a target optical response, the device structure **X** at the design area can be updated by gradient decent. The gradient of optical response with respect to the geometry structure is

(5) $$\frac{dy}{dX}=\frac{dy}{dE}\cdot \frac{dE}{d\varepsilon }\cdot \frac{d\varepsilon }{dX}.$$

The terms d ***y***/d**E** and d ***ɛ***/*d***X** are analytical, while the non-analytical middle term d**E**/*d* ***ɛ*** can be calculated by adjoint method with two simulations. In this way, all the parameters **X** can be updated with a learning rate *α* as

(6) $$X^{\left(new\right)}=X^{\left(old\right)}+\alpha \frac{dy}{dX}.$$

### 2.2 Minimal feature size control in Fourier domain

**Figure 1:**
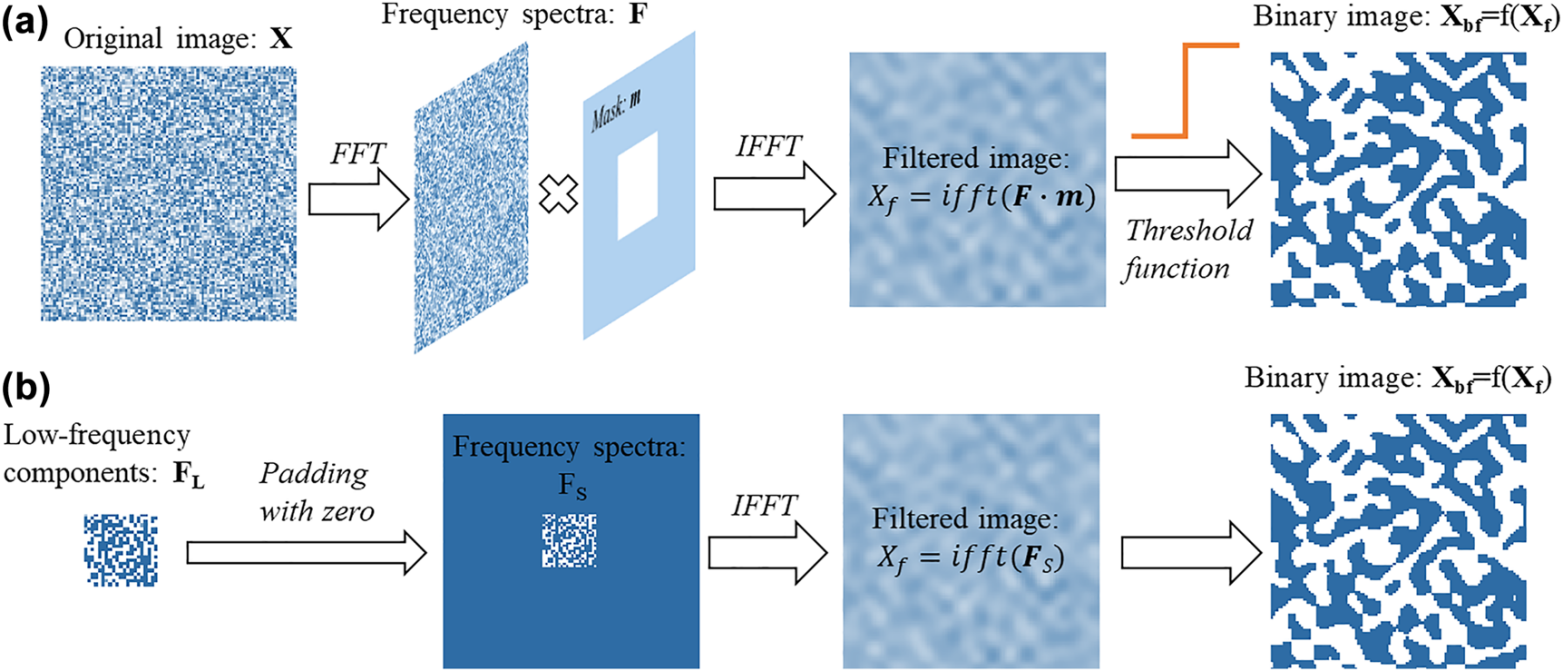
Minimal feature size control methods. (a) Control the minimal feature size by filtering out the high frequency components in the Fourier domain. (b) Utilizing the low-frequency components to reconstruct the real structure.

Even though the proposed device has a DOF of 201 × 101, not all of the combinations are able to be fabricated. In other words, some pixels do not contribute significantly to final device performance. A popular way to control the minimal feature size is to use a filter to remove small features throughout optimization [18], which can be realized either in real space or in frequency domain. In space domain, the original random image is convolved with a kernel to filter out noises from the original image. Then, a threshold function is applied to binarize the filtered image. As a result, isolated features are reduced. In frequency domain, the high-frequency components represent the rapid changes in an image, i.e., the noise and details, while the low-frequency components represent the main part of the image. If high frequency components of an image are filtered out, it would also be smoothed. As shown in Figure 1(a), the original image is converted to the Fourier domain F by fast Fourier transform (FFT). Then, a mask m which only allows the low-frequency components to pass is multiplied with F. By applying inverse fast Fourier transform (IFFT) and an activation function, an image with most pixels gathered is also obtained, while the convolution processing in space domain is replaced by the multiplication in Fourier domain.

Further, instead of taking the whole image in the real space as the design parameters, we utilize the lowfrequency components exclusively to reconstruct the image. As shown in Figure 1(b), the low-frequency components **F**_**L**_ is padded with zero to ensure the size of the frequency spectra **F**_**S**_ is the same as the image in real space. After IFFT and binarization, the reconstructed structure is the same as Figure 1(a), while the design parameters are the low-frequency components **F**_**L**_ other than the whole image **X** the real space. This processing method can be both used for controlling the minimal feature size as well as reducing the redundant DOF.

### 2.3 DNN-assisted topology optimization in Fourier domain

**Figure 2:**
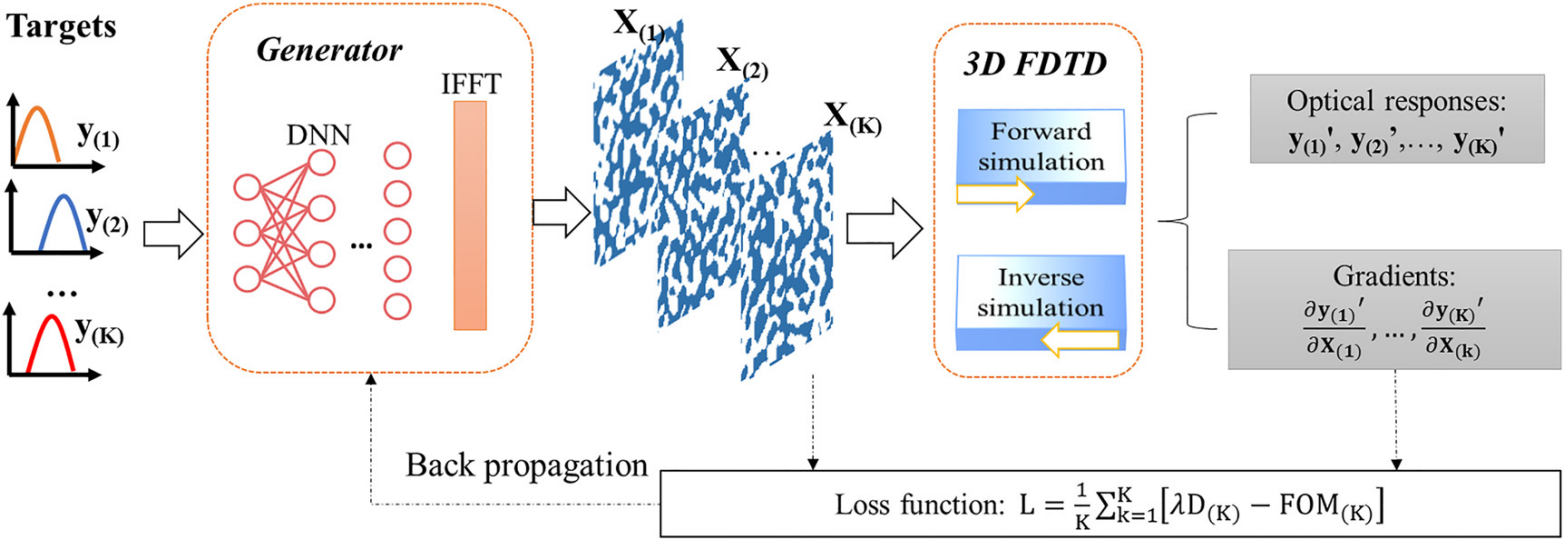
Schematic of the proposed DNN-assisted topology optimization in Fourier domain for multi-task optimization.

Topology optimization is an efficient way of optimizing device with large DOF. However, based on gradient descent algorithm, it is prone to fall into local optimal. Besides, it only works for single-task optimization. Fortunately, DNNs bridging the device geometry with optical response can be used to compensate the disadvantages of traditional topology optimization. Traditionally, DNNs are data samples dependent. If the DOF is too high, the network has to be scaled up and the required number of training data samples will amount. As shown in Figure 2, we propose a new DNN-assisted topology optimization method which efficiently combines the advantages of topology optimization and deep neural network. Since only the low-frequency components in Fourier domain are considered, the minimal feature size can be controlled easily while the redundant DOF is reduced to make the network easier to converge in training.

The DNN consists of 4 fully-connected layers with 100, 512, 1024 and 870 neurons in each layer. The input of the DNN is the target optical response ***y***. The activation functions of the middle layers are LeakyReLU. The output **O** of the DNN is separated into **O**_**1**_ and **O**_**2**_, which represents the real and the imaginary parts of low-frequency components in Fourier domain, respectively. By combing them as complex numbers and reshaping them to two-dimensional data, the low-frequency components **F**_**L**_ in Fourier domain are obtained. The high-frequency components **F**_**H**_ in Fourier domain are replaced by zero to get the full spectra **F**. After the IFFT, we can get the image **I** in space, which is further activated by Tanh function to obtain the device structure **X**. The predicted device structure **X** is simulated by a 3D FDTD solver to calculate the actual optical response ***y***’. The FOM of *k*th predicted device is defined as

(7) $$FOM_{\left(k\right)}=1-{\left|\overset{\lambda_{\max}}{\underset{\lambda =\lambda_{\min}}{\int }}\frac{{\left|y_{\left(k\right)}\left(\lambda \right)-{y_{\left(k\right)}}^{\prime }\left(\lambda \right)\right|}^{p}}{\lambda_{\max}-\lambda_{\min}}d\lambda \right|}^{\frac{1}{p}},$$

where *λ* is the working wavelength. The parameter *p* is a normalization constant, which is set as 2 in our optimization for the consideration of calculation speed and avoiding large prediction error.

Apart from the FOM, the discreteness, i.e., the state of binarization, is also an important metric of final generated device. The discreteness of *k*th device is defined as

(8) $$D_{\left(K\right)}=-\left|X_{\left(k\right)}\right|\cdot \left(2-X_{\left(k\right)}\right).$$

When all the pixels in **X** are binarized with value of −1 or 1, D gets the smallest value as −1. Therefore, the loss function of *K* generated devices can be expressed as

(9) $$L=\frac{1}{K}\overset{K}{\underset{k=1}{\sum }}\left(\lambda D_{\left(k\right)}-FOM_{\left(k\right)}\right),$$

where the hyper-parameter *K* is the number of generated devices at each training epochs. It is also referred to as the mini-batch size for the network training [32]. The set of batch size is problem-related. For simulator-based network training, the required time for the whole training procedure mainly comes from simulation. Therefore, the choose of batch size is determined by the available computation resources to ensure fastest parallel simulation. For our computer, we set *K* to be 12. Another hyper-parameter *λ* is used to control the preference of performance or binarization progress. It is assigned a gradually increasing value as

(10) $$\lambda =\frac{n}{N},$$

where *n* is the current *n*th training epoch and *N* is the total training epochs. The hyper-parameter *λ* is a small value at the beginning to make sure the device achieves high performance and then it is increased to ensure the optimized device is totally binary.

With above loss function *L*, the DNN can be trained by backpropagation. The gradients of *L* with respect to parameters in DNN are calculated as

(11) $$\begin{array}{ll} \frac{\partial L}{\partial w_{m}} & =\frac{1}{K}\overset{K}{\underset{k=1}{\sum }}\frac{\partial L}{\partial X_{\left(k\right)}}\cdot \frac{\partial X_{\left(k\right)}}{\partial w_{m}}\\ & =\frac{1}{K}\overset{K}{\underset{k=1}{\sum }}\left(\lambda \frac{\partial D_{\left(k\right)}}{\partial X_{\left(k\right)}}-\frac{\partial FOM_{\left(k\right)}}{\partial X_{\left(k\right)}}\right)\cdot \frac{\partial X_{\left(k\right)}}{\partial w_{m}}. \end{array}$$

Where the first term ∂*X*_(*k*)_/∂*w*_*m*_ is analytical and the third term ∂*X*_(*k*)_/∂*w*_*m*_ determined by the DNN is also analytical. The non-analytical second term can be calculated as

(12) $$\begin{array}{ll} \frac{\partial FOM_{\left(k\right)}}{\partial X_{\left(k\right)}} & =-{\left|\overset{\lambda_{\max}}{\underset{\lambda =\lambda_{\min}}{\int }}\frac{{\left|y\left(\lambda \right)-y^{\prime }\left(\lambda \right)\right|}^{p}}{\lambda_{\max}-\lambda_{\min}}d\lambda \right|}^{\frac{1}{p}-1}\cdot \overset{\lambda_{\max}}{\underset{\lambda =\lambda_{\min}}{\int }}\\ & \;\times \frac{\;{\left|y\left(\lambda \right)-y^{\prime }\left(\lambda \right)\right|}^{p-1}\cdot \;\mathrm{sign}\left|y\left(\lambda \right)-y^{\prime }\left(\lambda \right)\right|}{\lambda_{\max}-\lambda_{\min}}\cdot \\ & \;\times \frac{\partial y\left(\lambda \right)}{\partial X_{\left(k\right)}}d\lambda . \end{array}$$

where the term ∂*y*(*λ*)/∂*X* can be calculated by Eq. (5) with the adjoint method.

## 3 Multi-task parallel optimization of wavelength filters

### 3.1 Problem setup

Integrated wavelength filters which selectively transmit certain wavelengths and block the others are essential for optical signal processing. The most widely used filters are low-pass, high-pass, band-pass and band-stop filters [33–36]. However, these proposed empirical-based structures in large footprint are for specific functions, while versatile wavelength filters are required for many applications.

**Figure 3:**
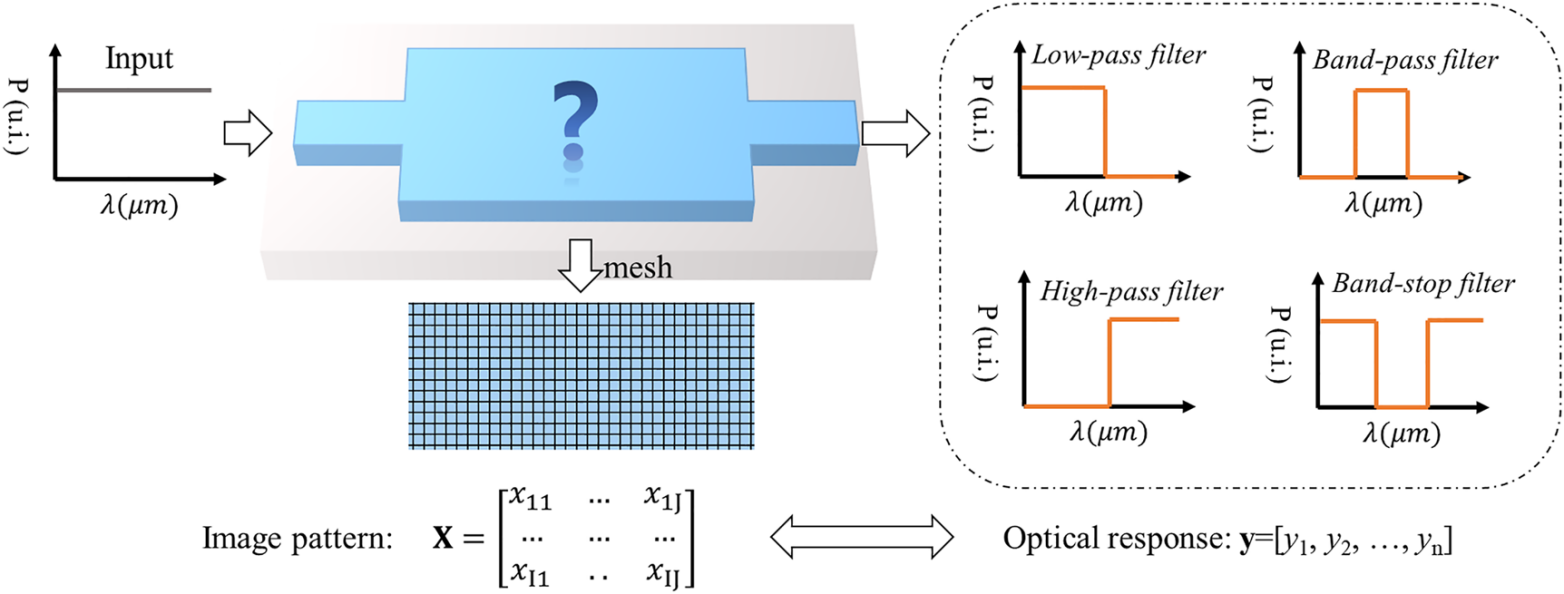
Schematic of the wavelength filter on SOI platform. Various transmission spectra can be achieved with different patterns in the middle slab region.

To increase the diversity of wavelength filters for different application scenarios, we propose a compact and general structure on a 220 nm thick standard SOI platform with silica cladding. As shown in Figure 3, with full spectrum input, the device will output various optical responses when the middle area is patterned with different images. The middle slab area is a 4 × 2 μm^2^ rectangle where each pixel is filled with either silicon or silica. The size of each pixel is 20 × 20 nm^2^ for the balance of simulation accuracy and optical efficiency. The middle slab area connects with two 500 nm wide waveguides for input and output.

For mathematical representation, the geometry structure of the middle area is presented as a binary matrix

(13) $$X=\left[\begin{array}{ccc} x_{1,1} & \ldots & x_{1,201}\\ \ldots & \ldots & \ldots \\ x_{\mathrm{101,1}} & \ldots & x_{\mathrm{101,201}} \end{array}\right]$$

where *x*_ij_ represents the pixel of *i*th row and *j*th column in the design area. The value of *x*_ij_ is 1 or −1 when it is filled with silicon or silica. The wavelength range of the proposed device is 1260–1360 nm, where 100 points are evenly sampled from the spectra. The optical response of the device is recorded as

(14) $$y=\left[y_{1},\;y_{2},\;\ldots ,\;y_{\mathrm{100}}\right]$$

where *y*_*k*_ represents the transmission efficiency at the *k*th wavelength point.

### 3.2 Setting the size of Fourier components

**Figure 4:**
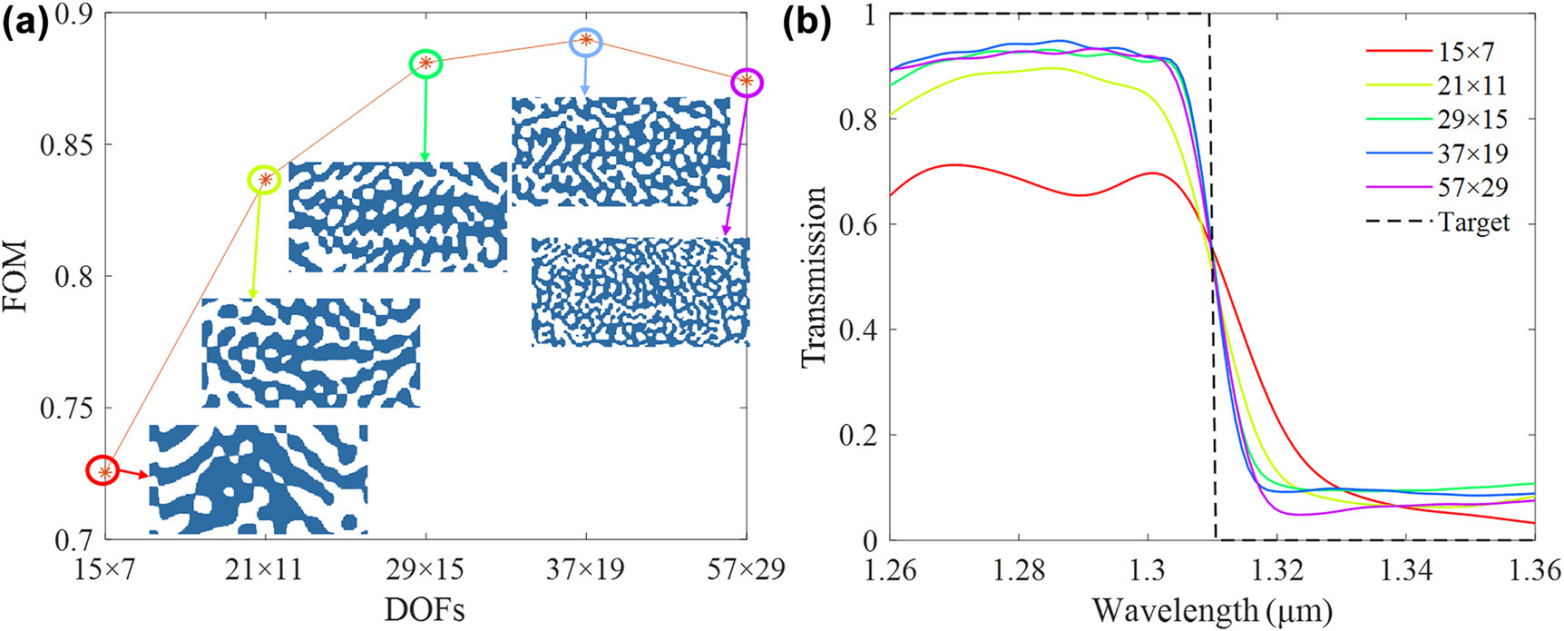
The investigation of impacts for different DOFs. (a) Optimized wavelength filters with five different DOFs as 15 × 7, 21 × 11, 29 × 15, 37 × 19 and 49 × 25. (b) Optical responses of five optimized wavelength filters with different DOFs, where the solid lines represent the simulated results and the dashed line represent the optical target.

In our proposed method, the size of low-frequency components, also referred as the DOF, determines how many details should be included for optimization. To investigate the effects of changing DOF, five wavelength filters are designed with DOFs of 15 × 7, 21 × 11, 29 × 15, 37 × 19 and 57 × 29, respectively. The final FOMs of optimized wavelength filters with different DOFs are shown in Figure 4(a), where inset images depict optimized structures. The solid curves in Figure 4(b) demonstrate optical responses of optimized wavelength filters with different DOFs, while the dashed line represents the optical target. Except for the DOF of 15 × 7, the rest devices are with similar optical responses. We also notice that when the DOF is too large such as 57 × 29, the performance will decrease. This is because the optimization is prone to fall into local optimal when the DOF is too large. Further increasing DOF will not improve the performance significantly, while the complexity of the geometry will increase dramatically. Therefore, we set the DOF to 29 × 15 for the rest of our optimization.

The choice of DOF, or in our case, the number of Fourier components, is based on trial and error. Quantitative estimation of optimization bounds as proposed recently [37] is promising to facilitate our design approach, although the application of such method in problems involving 3D FDTD simulations is still challenging [38]. It would be interesting to further explore the bound estimation of 3D problems in our future work, as a guidance to our inverse design procedures.

### 3.3 Multi-task optimization results

**Figure 5:**
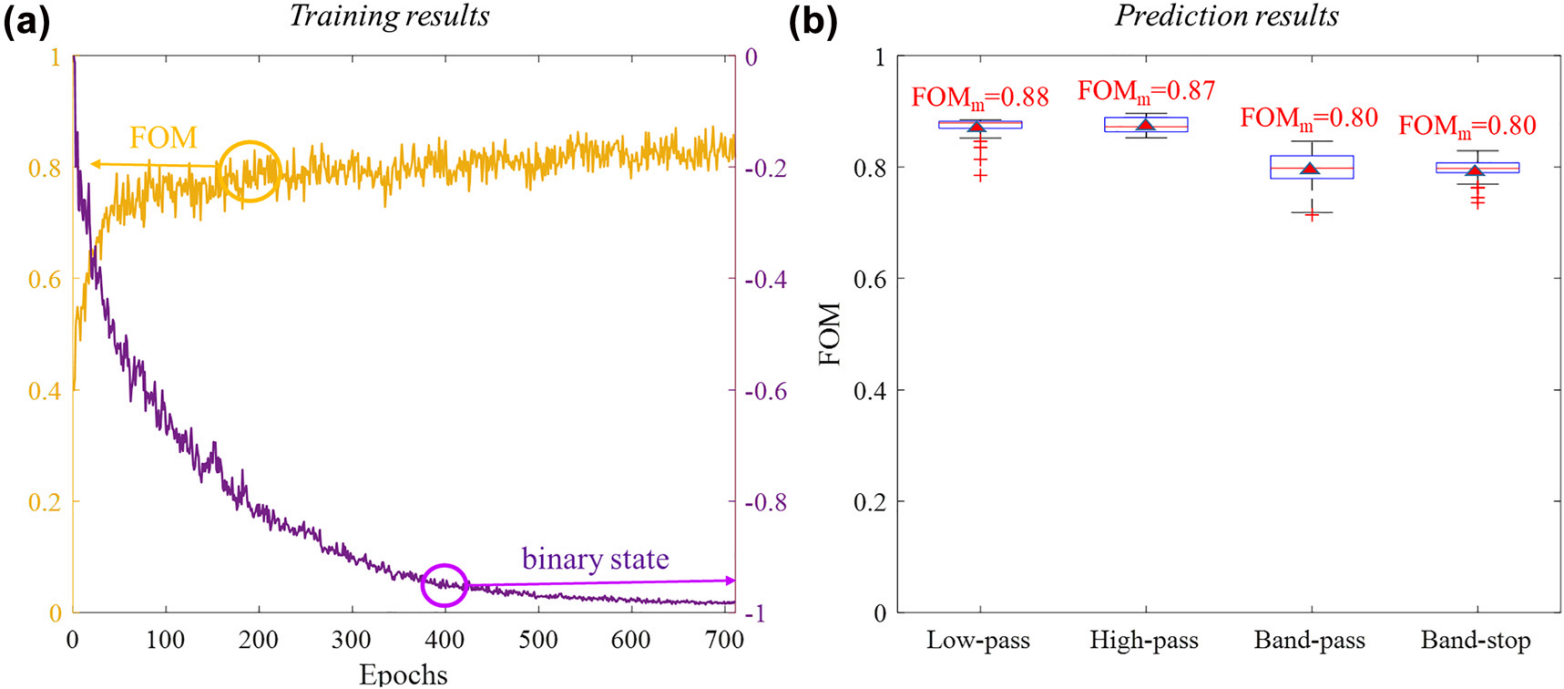
Multi-task optimization results of wavelength filters. (a) Mean FOM (yellow curve) and mean binary state (purple curve) of randomly generated optical targets at each training epoch. (b) Boxplot of the FOMs of predicted devices by the trained DNN for randomly generated optical targets.

To ensure the diversity of samples during training process, wavelength filters with random threshold wavelengths and random filter widths are generated as the targets for each epoch. For the balance of network training efficiency and simulation speed, 12 randomly generated targets are input to the DNN for parallel calculation. The training results are shown in Figure 5(a), where the yellow curve and purple curve represent the mean FOM and mean binary state at each training epoch, respectively. As the targeted spectra at each epoch are quite different, the training curves have many ripples. After 711 training epochs, the training curve stabilized. The mean FOM is converged to 0.82 and the mean binary state is close to −1. Overall, it takes 17,064 simulations for this multi-task network training. It takes less than two weeks to finish the multi-task network training on our computer (Intel(R) Xeon(R) Platinum 8171 M CPU). The training time can be further reduced with more computing resources, as parallel simulations are supported for our proposed multi-task optimization method. It can also be reduced by limiting the number of random targets at each epoch, depending on the scale of the devices required for optimization.

To test the performance of the trained DNN, 100 low-pass, high-pass, band-pass and band-stop wavelength filters are randomly generated as the targets. The trained DNN predicts their corresponding device structures within a second. To measure the performance of generated devices, these predicted structures are further simulated by 3D FDTD method to calculate their actual transmission spectra. The FOMs of four kinds of predicted wavelength filters are shown in the box plot in Figure 5(b). The mean FOMs of low-pass and high-pass filters are 0.88 and 0.87, while the mean FOMs of band-pass and band-stop filters are 0.80. Besides, for all the randomly generated optical targets, the trained DNN could predict their corresponding devices with FOMs of larger than 0.72, even for the worst cases.

**Figure 6:**
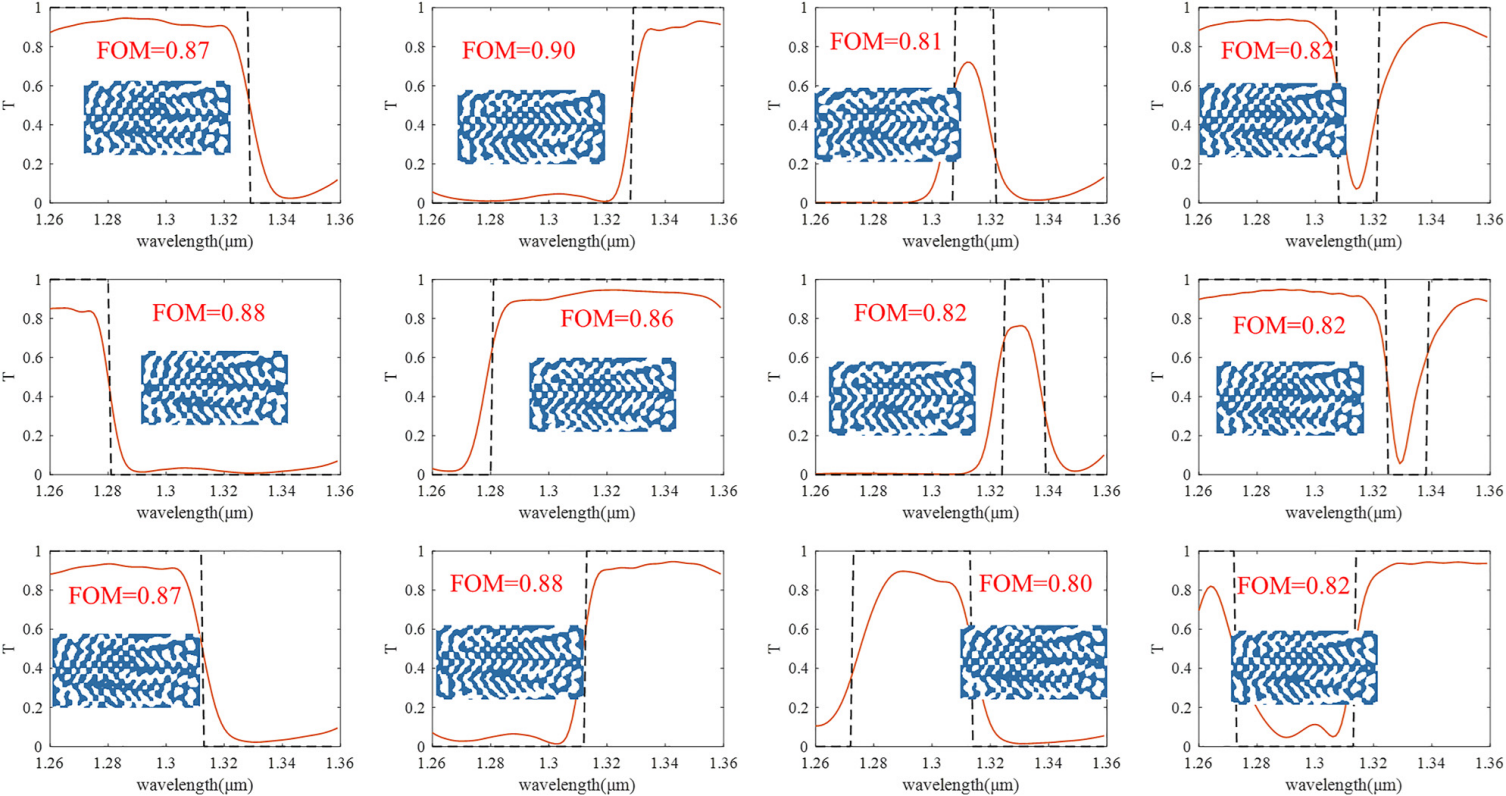
Illustration of 12 predicted wavelength filters. The dashed lines represent the randomly generated optical targets, while the solid lines represent the actual optical responses of predicted devices by the trained DNN. The insect images of each subplot figure show the predicted device structures.

For clearer illustration, 12 predicted wavelength filters are shown in Figure 6. The dashed lines represent the target spectra, while the solid lines represent the real transmission spectra of the devices predicted by our trained DNN. The inset images of each subplot figure are the predicted device structures. Figure 6 suggests that the spectra of the generated devices are very close to design targets. More importantly, for our method the device performance can be further improved by continuously fine-tuning the DNN, while for traditional data-driven DNNs the network will lose their ability for prediction if the target is too far away from the prepared training dataset.

## 4 Discussions

### 4.1 Achieving additional design targets by transfer learning

**Figure 7:**
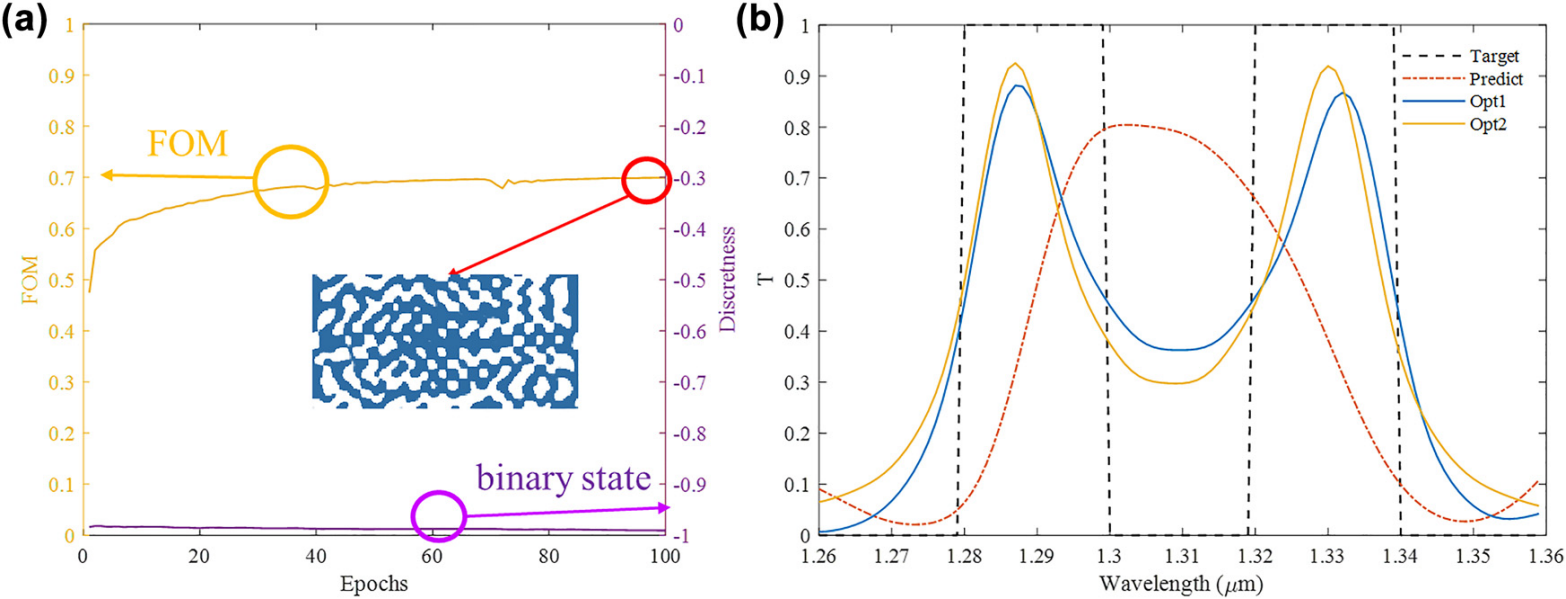
Optimization of a new wavelength filter with two opening windows. (a) Optimization procedure with pre-trained DNN, where yellow line represents the FOM and purple line represents the discreteness. The inset figure shows the further optimized structure. (b) Black and orange dash lines represent the target and predicted optical spectra, while blue (Opt1) and yellow (Opt2) solid lines represent optimized spectra with and without pre-trained DNN.

For most DNN-based inverse design, if a targeted optical response is quite different from the training data samples, the performance of predicted device is not guaranteed. However, with our method, completely new targets can be optimized based on the current network without the need of an optimization round from scratch, as the DNN pre-trained by multi-task optimization can be used just like transfer learning. For example, spectra with two filtering windows never exist in our training targets. As such target is too far away from the original training samples, the spectrum of predicted device resembles the original training samples with only one filtering window, as illustrated by red dashed line in Figure 7(b). As shown in Figure 7(a), the pre-trained network is further train with the new design target. Since the pre-trained DNN has already learn to binarize the predicted structure, we can find the discreteness is always close to one in all training epochs. After 100 epochs of further training with new optical response, the FOM can be improved from 0.46 to 0.70. The optimized spectrum denoted by blue solid line in Figure 7(b) is much similar to our target. For comparison, we also optimized such an optical filter without the pre-trained DNN. The optimized FOM is also around 0.7 and the spectrum is denoted as yellow solid line in Figure 7(b), which is quite similar to the optimized results by further learning with pre-trained DNN. However, it takes 400 epochs to get such optimization result without the pre-trained DNN. It suggests even though certain optical target is quite different from the training samples, it can be further optimized with significantly reduced training epochs using our pre-trained generator.

### 4.2 Multi-stage optimization

**Figure 8:**
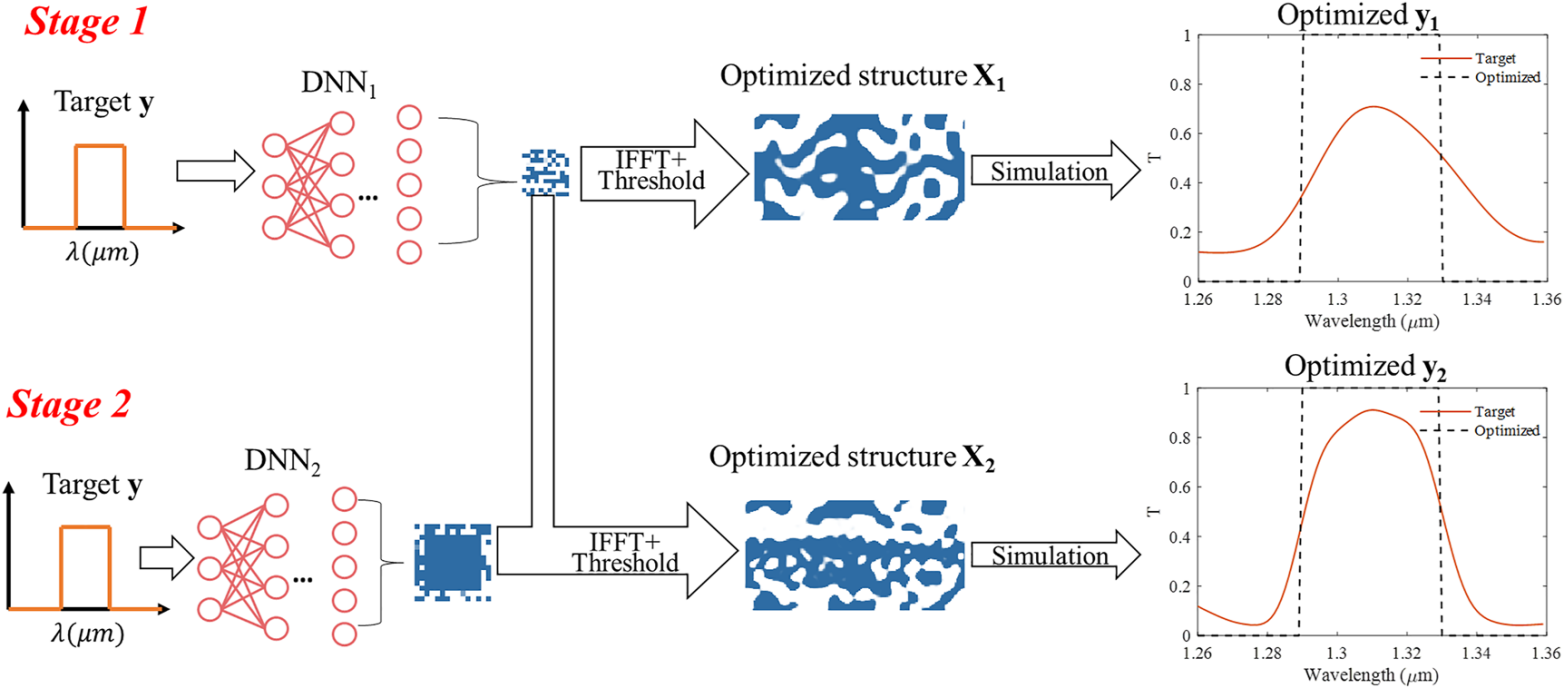
Schematic of multi-stage optimization procedure. In stage 1, the optical target is mapped to the low-frequency components in fourier space by a simple DNN_1_. In stage 2, the optical target is mapped to outer Fourier components by another simple DNN_2_. The right pictures show the optimized structures **X**_**1**_, **X**_**2**_ and their corresponding wavelength spectra **y**_**1**_, **y**_**2**_.

Our proposed method can also be extended for multi-stage optimization. When the design DOF is large, reusing the findings of simulations with fewer Fourier components in simulations with more Fourier components can be an effective way to reduce the complexity of the single DNNs and thus speed up the convergence of network training. For example, we implement the multi-stage optimization in our optical wavelength filters design as shown in Figure 8. In stage 1, the optical target (band pass filter spectrum) is mapped to the 15 × 7 low-frequency components in Fourier space by DNN_1_. The optimized structure in stage 1 is shown as **X**_**1**_ and the optimized spectrum is represented in **y**_**1**_ as the orange curve. Due to the lack of enough DOF, the FOM is only 0.67. In stage 2, the optimized low-frequency components in stage 1 are maintained. Meanwhile, the optical target is mapped to the outer layer Fourier components by DNN_2_, which is then combined with those optimized low-frequency components to form the enlarged 29 × 15 components to reconstruct the final device structure. After such optimization and component expansion, the final optimized structure is shown as **X**_**2**_, which includes more details compared to **X**_1_. The optimized wavelength spectrum in stage 2 is represented as the orange curve in **y**_**2**_, whose FOM is increased to 0.80.

### 4.3 Adaptation to the optimization of an on-chip single-photon source

**Figure 9:**
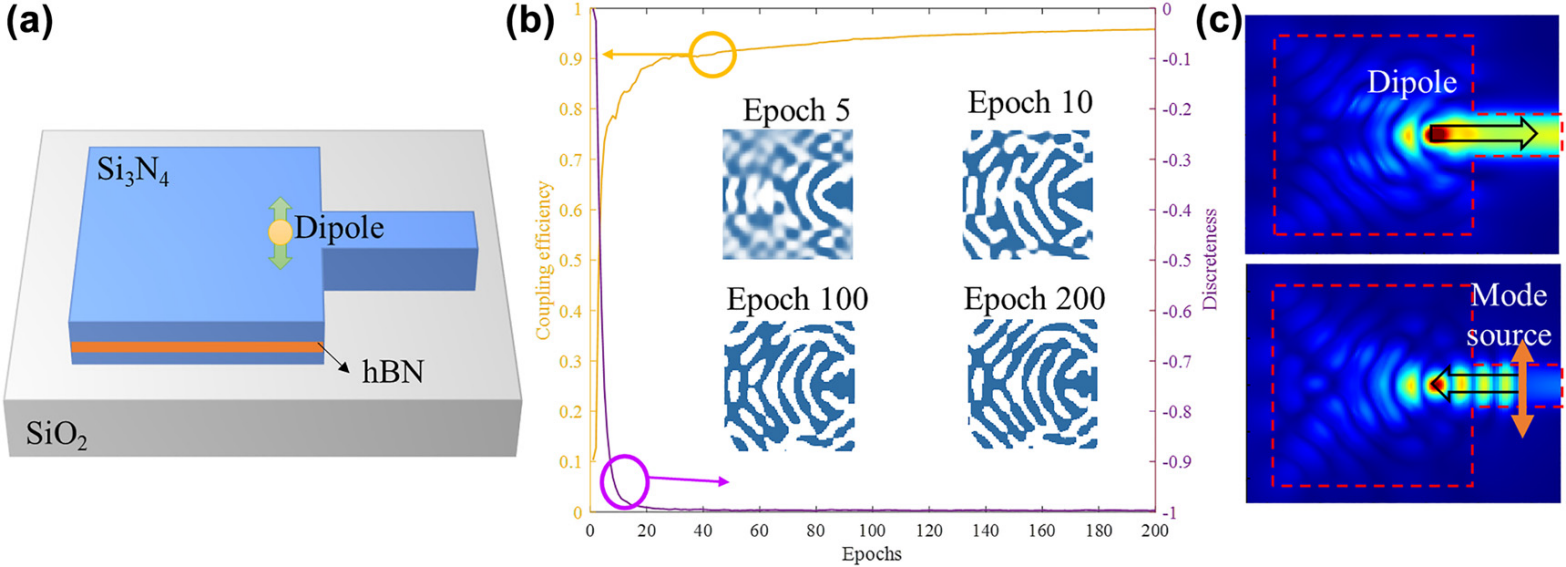
Optimization of single photon emission. (a) The schematic of embedded Si_3_N_4_-hBN hybrid cavity on a SiO_2_ substrate [39], where the square aera is also the design area. (b) Optimization procedure for high coupling efficiency with our method, where the inset figures show the generated structure at different training epochs. (c) Simulated electric fields with dipole input (upper one) and mode source input (lower one) of the optimized device structure.

Apart from the design of wavelength filters, the proposed method can also be extended for the optimization for other nanophotonic devices. For example, Omer et al. demonstrated a topology-optimized waveguide coupler for single-photon source [39]. It can also be optimized by our DNN-based optimization method. The embedded Si_3_N_4_-hBN hybrid cavity on a SiO_2_ substrate is shown in Figure 9(a). The hybrid 2 × 2 μm^2^ square area is the design area. The mesh accuracy here is also set as 20 × 20 nm^2^ for the balance of computation speed and simulation accuracy. The size of Fourier components is set to be 15 × 15 to enable enough design DOF and to filter out those small features. As the optical target in this case is only 1, the generator is supposed to map 1 input to 2 × 15 × 15 outputs. During training, it is prone to have the gradient exploding problem. To solve this problem, the 1 optical target is represented by a vector with 100 dimensions through an embedding layer first. Then the network and other hyper-parameters are set to be the same as Section 3. As it is a single-task optimization, the training curve is smooth as shown in Figure 9(b). The coupling efficiency is converged to 0.95 and the optimized device is totally discrete after 200 training epochs. The inset figures in Figure 9(b) illustrate the device structures at different training epochs. Along the optimization procedure, the generated device structure evolves toward the shape of a Bragg-reflector-like structure, as such structure could enable highly efficient field redirection towards the waveguide. The electrical fields of the optimized device structure are shown in Figure 9(c), where light is well coupled to the waveguide. This example suggests that our proposed method can be generalized to other device optimization problems, including those with high field intensities.

## 5 Conclusions

In conclusion, we proposed a DNN-assisted topology optimization method in Fourier domain for the design of integrated wavelength filters. The targeted optical responses are input to the DNN, where the low-frequency component components in Fourier domain are output. After zero-padding, IFFT and activation function, the device structure patterns in real space can be reconstructed. By forward and adjoint 3D FDTD simulations, the loss gradients of generated device structures are calculated, which can be used for the training of DNN. Taking the low-frequency Fourier components as DOF helps control the minimal feature size of generated devices. It can also reduce the redundant DOF to make the DNN prone to converge. This DNN-driven topology optimization method can be used for concurrent optimizations of multiple wavelength filters, where randomly generated wavelength filters are input as the targets for network training. After 711 training epochs, the mean FOM is converged to around 0.82. The trained DNN can also be further utilized for the design of totally different wavelength filters through transfer learning, where fewer optimization epochs would be required compared to optimization from scratch. What is more, the proposed method can also be used for multi-stage optimization with simpler DNNs at each stage. To demonstrate the generalizability of our method, we also adapt it to the design of waveguide-coupled single-photon source with high coupling efficiency. Our proposed method paves a new way for the design of free-form and compact nano-photonic devices.

## References

[1] Thomson D., Zilkie A., Bowers J. E. (2016). Roadmap on silicon photonics. J. Opt..

[2] Ferreira de Lima T., Shastri B. J., Tait A. N., Nahmias M. A., Prucnal P. R. (2017). Progress in neuromorphic photonics. Nanophotonics.

[3] Qiang X., Zhou X., Wang J. (2018). Large-scale silicon quantum photonics implementing arbitrary two-qubit processing. Nat. Photonics.

[4] Mao S., Cheng L., Zhao C., Khan F. N., Li Q., Fu H. (2021). Inverse design for silicon photonics: from iterative optimization algorithms to deep neural networks. Appl. Sci..

[5] Yao K., Unni R., Zheng Y. (2019). Intelligent nanophotonics: merging photonics and artificial intelligence at the nanoscale. Nanophotonics.

[6] Molesky S., Lin Z., Piggott A. Y. (2018). Inverse design in nanophotonics. Nat. Photonics.

[7] Yu Z., Cui H., Sun X. (2017). Genetic-algorithm-optimized wideband on-chip polarization rotator with an ultrasmall footprint. Opt. Lett..

[8] Liu Z., Liu X., Xiao Z. (2019). Integrated nanophotonic wavelength router based on an intelligent algorithm. Optica.

[9] Mao S., Cheng L., Zhao C., Fu H. (2021). Ultra-broadband and ultra-compact polarization beam splitter based on a tapered subwavelength-grating waveguide and slot waveguide. Opt. Express.

[10] Zhang Y., Yang S., Lim A. (2013). A compact and low loss Y-junction for submicron silicon waveguide. Opt. Express.

[11] Chen W., Zhang B., Wang P. (2020). Ultra-compact and low-loss silicon polarization beam splitter using a particle-swarm-optimized counter-tapered coupler. Opt. Express.

[12] Guan H., Ma Y., Shi R. (2014). Ultracompact silicon-on-insulator polarization rotator for polarization-diversified circuits. Opt. Lett..

[13] Cheng L., Mao S., Chen Z., Wang Y., Zhao C., Fu H. (2021). Ultra-compact dual-mode mode-size converter for silicon photonic few-mode fiber interfaces. Opt. Express.

[14] Piggott A. Y., Lu J., Lagoudakis K. G., Petykiewicz J., Babinec T. M., Vučković J. (2015). Inverse design and demonstration of a compact and broadband on-chip wavelength demultiplexer. Nat. Photonics.

[15] Mao S., Cheng L., Zhao C., Fu H. (2021). Coarse wavelength division (de)multiplexer based on cascaded topology optimized wavelength filters. Proc. CLEO.

[16] Jiang J., Fan J. A. (2019). Global optimization of dielectric metasurfaces using a physics-driven neural network. Nano Lett..

[17] Vercruysse D., Sapra N. V., Su L., Trivedi R., Vuckovic J. (2019). Analytical level set fabrication constraints for inverse design. Sci. Rep..

[18] Hammond A. M., Oskooi A., Johnson S. G., Ralph S. E. (2021). Photonic topology optimization with semiconductor-foundry design-rule constraints. Opt. Express.

[19] Wang K., Ren X., Chang W., Lu L., Liu D., Zhang M. (2020). Inverse design of digital nanophotonic devices using the adjoint method. Photon. Res..

[20] Khoram E., Qian X., Yuan M., Yu Z. (2020). Controlling the minimal feature sizes in adjoint optimization of nanophotonic devices using b-spline surfaces. Opt. Express.

[21] So S., Badloe T., Noh J., Bravo-Abad J., Rho J. (2020). Deep learning enabled inverse design in nanophotonics. Nanophotonics.

[22] Hammond A. M., Camacho R. M. (2019). Designing integrated photonic devices using artificial neural networks. Opt. Express.

[23] Tang Y., Kojima K., Koike-Akino T. (2020). Generative deep learning model for inverse design of integrated nanophotonic devices. Laser Photon. Rev..

[24] Ren Y., Zhang L., Wang W. (2021). Genetic-algorithm-based deep neural networks for highly efficient photonic device design. Photon. Res..

[25] Mao S., Cheng L., Khan F. N. (2022). Inverse design of high-dimensional nanostructured 2×2 optical processors based on deep convolutional neural networks. J. Lightw. Technol..

[26] Long Y., Ren J., Li Y., Chen H. (2019). Inverse design of photonic topological state via machine learning. Appl. Phys. Lett..

[27] Gostimirovic D., Ye W. N. (2019). An Open-Source Artificial neural network model for polarization-insensitive silicon-on-insulator subwavelength grating couplers. IEEE J. Sel. Top. Quant. Electron..

[28] Melati D., Grinberg Y., Kamandar Dezfouli M. (2019). Mapping the global design space of nanophotonic components using machine learning pattern recognition. Nat. Commun..

[29] Liu Z., Zhu Z., Cai W. (2020). Topological encoding method for data-driven photonics inverse design. Opt. Express.

[30] Jiang J., Fan J. A. (2019). Simulator-based training of generative neural networks for the inverse design of metasurfaces. Nanophotonics.

[31] Lalau-Keraly C. M., Bhargava S., Miller O. D., Yablonovitch E. (2013). Adjoint shape optimization applied to electromagnetic design. Opt. Express.

[32] Goodfellow I., Bengio Y., Courville A. (2016). *Deep Learning*.

[33] Xu P., Zhang Y., Zhang S., Chen Y., Yu S. (2020). Scaling and cascading compact metamaterial photonic waveguide filter blocks. Opt. Lett..

[34] Xu X. B., Guo X., Chen W. (2019). Flat-top optical filter via the adiabatic evolution of light in an asymmetric coupler. Phys. Rev. A.

[35] Magden E. S., Li N., Raval M. (2018). Transmissive silicon photonic dichroic filters with spectrally selective waveguides. Nat. Commun..

[36] Huang Q., Jie K., Liu Q., Huang Y., Wang Y., Xia J. (2016). Ultra-compact, broadband tunable optical bandstop filters based on a multimode onedimensional photonic crystal waveguide. Opt. Express.

[37] Molesky S., Chao P., Mohajan J., Reinhart W., Chi H., Rodriguez A. W. (2022). T-operator limits on optical communication: metaoptics, computation, and input-output transformations. Phys. Rev. Res. Int..

[38] Chao P., Strekha B., Kuate Defo R., Molesky S., Rodriguez A. W. (2022). Physical limits in electromagnetism. Nat. Rev. Phys..

[39] Yesilyurt O., Kudyshev Z. A., Boltasseva A., Shalaev V. M., Kildishev A. V. (2021). Efficient topology-optimized couplers for on-chip single-photon sources. ACS Photonics.

